# The embryological basis of subclinical hypertrophic cardiomyopathy

**DOI:** 10.1038/srep27714

**Published:** 2016-06-21

**Authors:** Gabriella Captur, Carolyn Y. Ho, Saskia Schlossarek, Janet Kerwin, Mariana Mirabel, Robert Wilson, Stefania Rosmini, Chinwe Obianyo, Patricia Reant, Paul Bassett, Andrew C. Cook, Susan Lindsay, William J. McKenna, Kevin Mills, Perry M. Elliott, Timothy J. Mohun, Lucie Carrier, James C. Moon

**Affiliations:** 1 UCL Biological Mass Spectrometry Laboratory, Institute of Child Health and Great Ormond Street Hospital, 30 Guilford Street, London, UK; 2 Cardiovascular Division, Brigham and Women’s Hospital, Boston MA, USA; 3 Department of Experimental Pharmacology and Toxicology, Cardiovascular Research Center, University Medical Center Hamburg-Eppendorf, Hamburg, Germany; 4 DZHK (German Center for Cardiovascular Research), partner site Hamburg/Kiel/Lübeck, Hamburg, Germany; 5 Institute of Genetic Medicine, Newcastle University, Newcastle, UK; 6 INSERM U970, Paris Cardiovascular Research Center—PARCC, Paris, France; 7 The Francis Crick Institute Mill Hill Laboratory, The Ridgeway, Mill Hill, London, UK; 8 UCL Institute of Cardiovascular Science, University College London, Gower Street, London, UK; 9 University of Bordeaux, CHU de Bordeaux, CIC1401, Bordeaux, France; 10 Biostatistics Joint Research Office, University College London, Gower Street, London, UK; 11 Barts Heart Center, The Cardiovascular Magnetic Resonance Imaging Unit and The Center for Rare Cardiovascular Diseases Unit, St Bartholomew’s Hospital, West Smithfield, London, UK

## Abstract

Hypertrophic cardiomyopathy (HCM) is caused by mutations in sarcomeric proteins, the commonest being *MYBPC3* encoding myosin-binding protein C. It is characterised by left ventricular hypertrophy but there is an important pre-hypertrophic phenotype with features including crypts, abnormal mitral leaflets and trabeculae. We investigated these during mouse cardiac development using high-resolution episcopic microscopy. In embryonic hearts from wildtype, homozygous (HO) and heterozygous (HET) *Mybpc3*-targeted knock-out (KO) mice we show that crypts (one or two) are a normal part of wildtype development but they almost all resolve by birth. By contrast, HO and HET embryos had increased crypt presence, abnormal mitral valve formation and alterations in the compaction process. In scarce normal human embryos, crypts were sometimes present. This study shows that features of the human pre-hypertrophic HCM phenotype occur in the mouse. In an animal model we demonstrate that there is an embryological HCM phenotype. Crypts are a normal part of cardiac development but, along with the mitral valve and trabeculae, their developmental trajectory is altered by the presence of HCM truncating *Mybpc3* gene mutation.

Hypertrophic cardiomyopathy (HCM) is the commonest monogenic heart disease (estimated prevalence 1 in 500), predominantly caused by autosomal dominant mutations in sarcomere protein genes^1^,^2^. Over 350 individual *MYPBC3* mutations have been identified, representing 40–50% of all HCM mutations, making it the most frequently mutated gene in this disease (Fig. 1). The majority of *MYPBC3* mutations are predicted to encode truncated proteins that lack portions of the C-terminus.

The majority of HCM patients survive into young adulthood^3^ but there are subgroups with a higher risk of sudden cardiac death and heart failure^4^. HCM is defined by unexplained left ventricular hypertrophy (LVH). Except for some neonatal forms of HCM^5^,^6^,^7^ where infants carry bi-allelic mutations, LVH typically appears for the first time in adolescence or early adult life^8^. Ahead of this however, patients may express a distinct subclinical phenotype consisting of measurable architectural abnormalities. This includes the presence of multiple myocardial crypts^9^,^10^, abnormal trabecular patterning^11^ and anterior mitral valve leaflet (AMVL) elongation^12^. In addition, microvascular remodelling^6^ and markers of myocardial fibrosis^13^ and altered function^14^ (particularly hypercontractility) may occur. As family screening of first-degree relatives is recommended in HCM^15^,^16^, the detection of this phenotype may be helpful in cases where genotyping is not definitive. Specific disease-modifying therapies are being developed for HCM including subclinical disease^17^,^18^ so tracking the subclinical phenotype earlier in the disease may help treatment^19^. We have previously shown how the subclinical phenotype can predict HCM sarcomere gene mutation carriage^20^, but its etiology remains unknown.

There are some clues. The architectural features and its identification in small children^6^ suggests an origin during cardiogenesis pre-birth. The noncompaction^11^,^21^ implies incomplete embryological compaction^22^ and potentially the persistence of an embryonic cardiac state. How other subclinical HCM features (like crypts and AMVL elongation) arise, is unknown but suspected to be similar^23^.

We explored whether myocardial crypts were a normal feature of cardiac morphogenesis and disordered in HCM, looking also at trabeculae and the mitral valve. We studied the phenotypic impact of a *Mybpc3* sarcomere gene mutation on murine cardiac development using high-resolution episcopic microscopy^24^ (HREM). We imaged wildtype human neonatal and embryonic hearts to explore normal development and we used HREM to systematically examine the cardiac morphogenesis of wildtype and HCM mouse hearts. HREM visualizes the hearts in three-dimensions (3D) at high resolution. We used a *Mybpc3*-targeted KO (knock-out [KO]) mouse model of HCM^25^ Briefly, the model was based upon the targeted deletion of exons 1 and 2 from the endogenous *Mybpc3* sarcomere gene, containing the transcription initiation site. In the homozygous (HO) state, KO mice develop LVH, systolic dysfunction and histopathological features of HCM shortly after birth while heterozygous (HET) KO exhibit later asymmetric septal hypertrophy^25^ (ASH) more akin to usual human HCM^26^,^27^.

## Methods

All methods described were carried out in accordance with the approved relevant guidelines.

### Normal embryonic, fetal and neonatal human hearts

We obtained this scarce resource from libraries, and imaged them using micro-computerised tomography (CT), micro-magnetic resonance imaging (MRI), HREM or optical projection tomography^28^ (OPT) according to heart size (Supplementary Table 1), searching for crypts. We obtained two embryonic (CS20 and CS21) and one fetal (9 pcw) human heart (visually fixed in diastole), from the Joint MRC/Wellcome-Trust (grant # 099175/Z/12/Z) Human Developmental Biology Resource^29^ (HBDR, http://www.hdbr.org), with appropriate maternal written informed consent and approval from the Newcastle and North Tyneside NHS Health Authority Joint Ethics Committee. One fetal (19 pcw) and one neonatal human heart was obtained from University College London Institute of Child Health. Both are regulated by the UK Human Tissue Authority (HTA; www.hta.gov.uk) and operate in accordance with the relevant HTA Codes of Practice.

### Animals

All mice (*Mus musculus*) were handled in accordance with the Guide for the Care and Use of Laboratory Animals published by the US National Institutes of Health and with the approval of the MRC National Institute of Medical Research Ethical Review Panel. Procedures were in accordance with the German Law for the Protection of Animals and accepted by the Ministry of Science and Public Health of the City State of Hamburg, Germany (Nr. ORG 696). Both KO and wildtype mice (Supplementary Table 2) were maintained on a C57Bl/6J background at the University Medical Center Hamburg-Eppendorf. The other larger population of wildtype mouse embryos was obtained from NIMR:Parkes (a robust outbred strain) and the inbred strain C57BL/6J maintained at the MRC National Institute of Medical Research. For approximate embryo staging, detection of a vaginal plug was taken as gestation day 0.5 (E0.5).

Embryo hearts were isolated as previously described^30^. These were dehydrated, infiltrated with methacrylate resin and used for HREM analysis^24^. Each HREM image stack comprised up to1300 short axis images, produce by successive removal of 2 μm (E14.5-E16.5) or 3 μm (E18.5-P0) sections. Mice were staged according to gestational age (Embryonic days) such that E0-0.9 refers to the one-cell embryo stage, E14.5 (Carnegie stage 22 in the human) marks the time when ventricular septation is complete and a dense trabecular meshwork is established within the ventricular cavities, E18.5 is immediately before birth and P0, the neonatal mouse.

### Image processing and analysis of murine data

After optimization of grayscale mapping, HREM datasets were subscaled to 250 Mb for 3D volume-rendered reconstructions in OsiriX (an open source software package that permits interactive 3D visualisation; 64bit version) and scored by a single reader, blinded to murine group and stage, for the presence or absence of crypts on short-axis HREM stacks and cross-checked on 3D volumetric models of the lumens. The segmental location of crypts was noted relative to the American Heart Association 17-segment model. Linear caliper measurements of crypt depth and adjacent wall thickness were acquired (Table 1). Crypts ≥30% of adjacent wall thickness were considered for all subsequent analysis. Fractal analysis for trabecular complexity and relevant reproducibility and reslicing experiments have been previously described^22^. For LV wall thickness linear caliper measurements we extracted using a semi-automated radial spokes approach developed in-house (40° sectors superimposed upon the HREM short axis slices in a clockwise direction; implemented in Image J version 1.38×, National Institute of Health, Bethesda, MD, USA). All mitral valve measurements were made on the 3-chamber long-axis view (replicating the cardiac MRI method^12^). AMVL and PMVL length was measured using the open-poly region of interest tool while basal leaflet thickness was measured using linear calipers in OsiriX. Intra-observer variability was estimated for AMVL length and basal leaflet thickness (n = 35) in randomly-selected mouse embryo datasets by HREM. Intra- and inter-observer variability was estimated for myocardial crypt rulings (n = 40) in randomly-selected mouse embryo datasets by HREM using two readers blinded to each other’s rulings (GC and CO).

### Statistical analysis

Statistical analysis was performed in R programming language (version 3.0.1, The R Foundation for Statistical Computing) and illustrations reporting bullseye plots constructed in MATLAB^®^ (The MathWorks Inc., Natick, MA, USA, R2012b). Descriptive data are expressed as mean ± standard deviation (s.d.) except where otherwise stated. Distribution of data was assessed on histograms and using Shapiro-Wilk test. Categorical variables were compared by Fisher’s exact tests. Normally distributed continuous variables pertaining to wildtype, HO and HET KO were compared using analysis of variance (ANOVA) with Tukey’s post-hoc test. Intra- and inter-observer variability for crypt rulings was evaluated by the Cohen’s Kappa statistic. Intraclass correlation coefficient was used to compare variability of repeated mitral leaflet measurements. A two-factor fully cross-factored ANOVA model was used to compare FD between mutant and wildtype mouse populations. Covariate models (FD = *X|A* + *ε*, where epsilon signifies full replication) were constructed for the analysis of response FD to terms: *X|A*, where *A* was the fixed factor (WT, Het or HO KO mouse group), and covariate *X* the (numeric) relative slice position along the ventricle. A two-sided *P* value < 0.05 was considered significant.

## Results

### Singular crypts can be seen in the normal developing human heart

**Figure 2:**
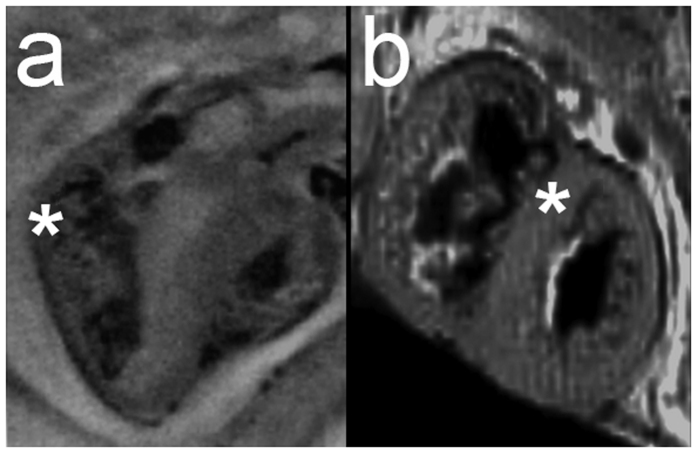
Crypts in developing human hearts. (**a**) A basal inferolateral crypt can be seen in this 3-chamber view of a CS21 human heart by OPT (asterix); (**b**) a mid anteroseptal crypt is seen in this short-axis view of a 9 pcw human fetus by MRI (asterix). CS = Carnegie stage; MRI = magnetic resonance imaging; OPT = optical projection tomography; pcw = post-coital week.

**Figure 3:**
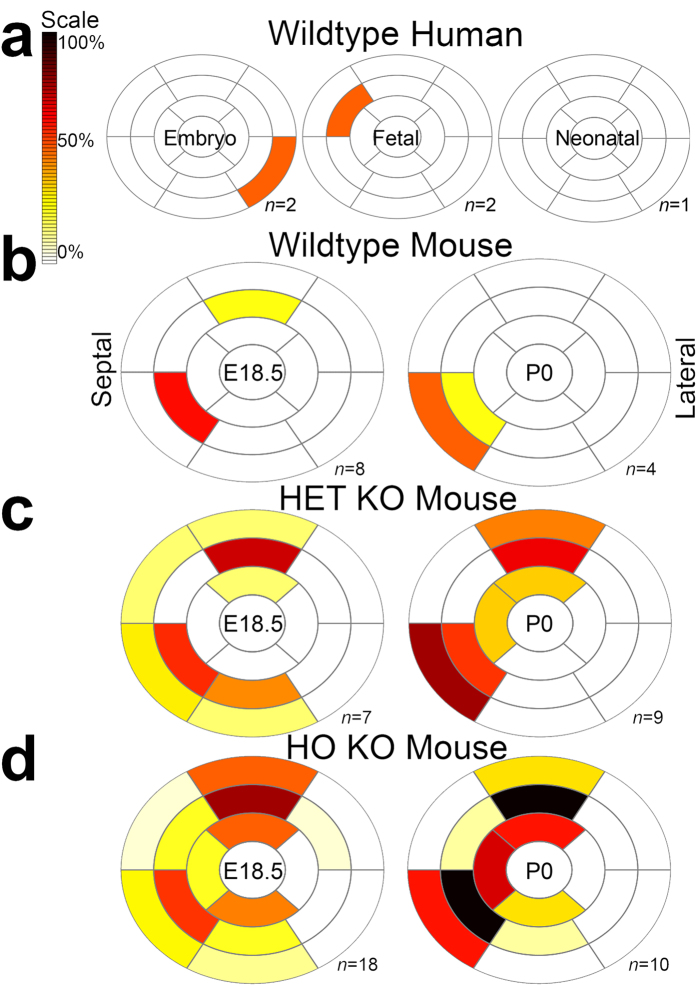
Myocardial crypts are congenital in humans and more abundant in embryonic HCM hearts. 17-Segment American Heart Association bullseye plots indicate the relative number of crypts per left ventricular segment as a percentage of the total number of hearts per group. (**a**) Normal human embryo hearts were studied at CS20 by HREM and CS21 by OPT, latter corresponding to E14.5 in the mouse. Fetal hearts were studied at 9 pcw by MRI and at 19 pcw by micro-CT. The neonatal human heart was studied by MRI. Compared to wildtype (**b**), HET and HO KO mouse mutants (**c**,**d**) showed a higher prevalence of multiple (≥2) crypts (by Fisher’s exact test): HET KO vs. wildtype at P0, *p* = 0.001; HO KO vs. wildtype at E18.5 and P0, *p* < 0.001 both. CT = computerized tomography; E = embryonic day; HREM = high-resolution episcopic microscopy; P0 = post-natal day 0. Other abbreviations as in Figs 1 and 2.

We show crypts could occur in the human embryo, observing one embryonic crypt at Carnegie Stage (CS) 21 and one fetal myocardial crypt at post-coital week (pcw) 9 (Figs 2 and 3a). No developing human heart exhibited multiple (≥2) crypts, an otherwise relatively common finding in adult patients with the *MYBPC3* mutation.

### Myocardial crypts are normal in the developing murine heart but few persist till birth

**Figure 4:**
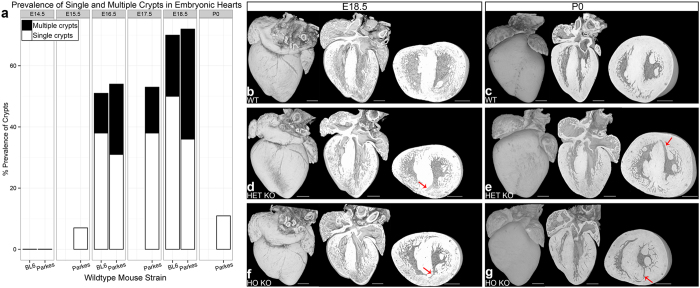
Myocardial crypts in the developing wildtype and HCM heart. (**a**) Crypt prevalence during development in wildtype mouse. Prevalence is calculated as a percentage of total number of hearts studied. Crypts are uncommon at E14.5 and E15.5, their prevalence increases between E16.5 and E18.5 but it falls again by P0 (when only 1 out of 9 NIMR:Parkes hearts exhibits a crypt). For C57BL/6J (BL6) and NIMR:Parkes (Parkes) strains respectively: E14.5, n = 13 and 15; E15.5, n = 0 and 14; E16.5, n = 8 and 13; E17.5, n = 0 and 12; E18.5, n = 10 and 14; P0, n = 0 and 9. (**b–g**) Heart sections by HREM from HO KO, HET KO and wildtype littermates at E18.5 (**b**,**d**,**f**) and P0 (**c**,**e**,**g**) showing respectively the whole-heart 3D reconstruction as well as the digitally eroded coronal and mid-ventricular short-axis view. Examples of deep myocardial crypts (red arrows in **d–g**) are highlighted in the slices from HET and HO KO (also Supplementary Videos 1 and 2). Crypts are deep myocardial defects that point into the myocardial wall typically around the RV insertion points. Scale bars, all 0.5 mm. The same magnification has been applied to all short-axis images – HO KO at P0 is visibly smaller than both wildtype and HET KO. WT denotes wildtype. Abbreviations as in Figs 1 and 3.

In wildtype murine embryo hearts (n = 108, Fig. 4a) across two strains (C57BL/6J, n = 31; NIMR:Parkes, n = 77) crypts are normal and ubiquitous in the embryo (total of 58 crypts counted in 108 hearts), making their first appearance in wildtype shortly after ventricular septation (embryonic day 15.5 [E]). These resembled adult human crypts (see Supplementary Figure 4). Prevalence increased beyond E15.5 and peaked at E18.5 (72% of NIMR:Parkes and 70% of C57BL/6J hearts exhibited at least 1 crypt at E18.5). By birth (P0), and with progressive myocardial thickening, crypts involuted so their prevalence dropped to 11% in wildtype. Intra- and inter-observer variability for crypt rulings was high (Cohen’s Kappa: intra-observer, 0.93; inter-observer, 0.84).

### Crypts are more abundant in the embryonic murine HCM heart

In HO KO, HET KO and wildtype littermates (n = 56 all on a C57BL/6J background, at both E18.5 and P0; HO KO n = 28, HET KO n = 16, wildtype n = 12) crypts are present in all at E18.5 but they persist to birth only in HO and HET KO mice, unlike wildtype (Figs 3c,d and 4b–g). When present, they are also more frequent (in total we counted 10 crypts in the wildtypes, 46 crypts in the HET KO and 98 crypts in the HO KO, Table 1). See Supplementary Videos 1 and 2.

### Abnormal development of the trabeculated myocardium in the embryo HCM heart

**Figure 5:**
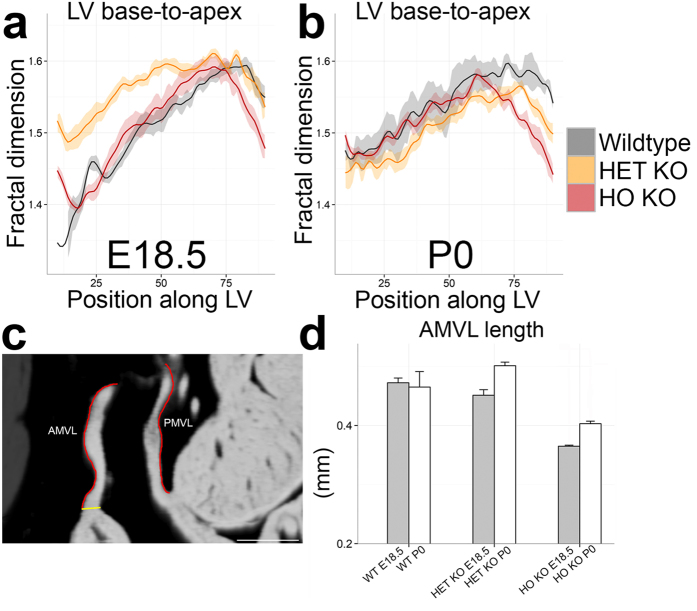
Trabecular and mitral differences between wildtype, HET and HO KO mice. Trabecular complexity (**a,b**) is increased in HET KO and reduced in HO KO. Solid lines: group means. Ribbons: upper/lower 95% confidence limits. FD^11^, an index of endocardial complexity (higher = more complex), was measured for each HREM slice. At E18.5 LV trabeculation in the basal half was higher in HET KO (FD mean ± s.d. in basal half: 1.540 ± 0.034) than in both wildtype (1.447 ± 0.055) and HO KO (1.462 ± 0.047, *p* < 0.0001 both, by ANOVA). At E18.5 and P0, the LV apex of HO KO (FD mean ± s.d. in the apical half at E18.5 and P0 respectively: 1.560 ± 0.028 and 1.535 ± 0.034) was less noncompacted compared to wildtype (1.565 ± 0.024 and 1.577 ± 0.012, *p* = 0.0003 and *p* < 0.0001 by ANOVA) and HET KO (1.590 ± 0.015 and 1.542 ± 0.016, *p* < 0.0001 and *p* < 0.05 by ANOVA). At E18.5 and P0 respectively for wildtype n = 8 and n = 4; HET KO n = 7 and n = 9; HO KO n = 18 and n = 10. (**c**) MV measurements (red: freehand length; yellow: linear caliper thickness) were performed on zoomed 3-chamber views from 3D curved multiplanar HREM reconstructions. (**d**) AMVL is shorter in HO KO than in wildtype and HET KO at E18.5 (*p* < 0.001, *p* < 0.01 respectively by one-way ANOVA) and shorter than HET KO at P0 (*p* < 0.05). AMVL basal thickness was reduced in HO KO compared to wildtype at E18.5 and P0 (*p* < 0.05 and *p* < 0.01 respectively). There were no differences in PMVL length/thickness between populations (Supplementary Table S6). Scale bar, 0.2 mm. Error bars, + sem. At E18.5 and P0 respectively for wildtype n = 8 and n = 4; HET KO n = 7 and n = 9; HO KO n = 18 and n = 10. A/PMVL = anterior/posterior mitral valve leaflet; FD = fractal dimension; LV = left ventricle; Other abbreviations as in Figs 1 and 3.

Trabeculation was quantified by fractal analysis^22^ (a higher value indicating more complexity^31^) in wildtype, HET and HO KO mice (n = 56 at E18.5 and P0; wildtype, n = 12; HET n = 16, HO n = 28, all on a C57BL/6J background). At E18.5 the LV of HET KO is less compacted than wildtype (Fig. 5a), normalising by P0. By contrast however the apex of HO KO was more compacted (smoother) than wildtype at all stages (Fig. 5a,b). The RV was similar - Supplementary Figure 1.

### HET KO mouse embryos do not show mitral valve leaflet elongation

We measured AMVL and posterior MVL (PMVL) length and basal leaflet thickness (Fig. 5c) in HCM and wildtype hearts at E18.5 and P0 in a total of 56 mice at E18.5 and P0 (wildtype, n = 12; HET KO n = 16, HO KO n = 28, all on a C57BL/6J background). There was no difference in AMVL length or thickness between HET KO and wildtype (Fig. 5d) even after adjusting for LV cavity size. We measured a shorter AMVL in HO KO mice at E18.5 than in both wildtype and HET KO that persisted after adjusting for LV cavity size at E18.5 (for HO KO compared to wildtype; *P* < 0.01, one-way ANOVA with Tukey correction). The AMVL of HO KO was also thinner than of wildtype at E18.5 and P0 but not after adjusting for LV cavity size (*P* = 0.99 and *P* = 0.16 respectively). There were no differences in absolute and cavity-size-adjusted PMVL measurements between wildtype, HET and HO KO. Intraclass correlation coefficients for repeated AMVL length and basal thickness measurements were high: 0.93, 95% confidence interval (CI) 0.90–0.96 and 0.91, 95% CI 0.87–0.95 respectively.

### Subtle ASH is normal during development but exaggerated in the HCM mice

**Figure 6:**
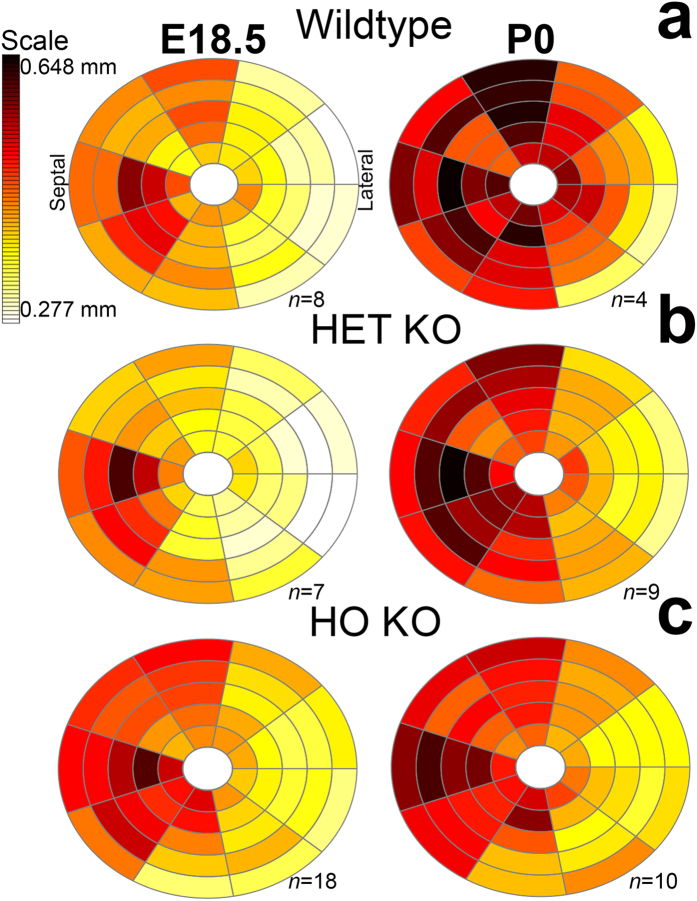
HCM KO mouse shows reduced LV wall thickness. Bullseye plots with 45 LV myocardial segments (9 radially at 5 levels from base to apex). Segment colours represent group means for wall thickness relative to the maximum/minimum value registered across all groups. HO KO mice (**c**) initially show higher total basal and septal-only basal wall thickness than wildtype ((**a**) at E18.5, *p* = 0.01 and *p* = 0.013 by one-way ANOVA with Tukey correction) but by P0 absolute values for basal to mid anteroseptal wall thickness were significantly lower in HO KO than in both wildtype (*p* < 0.01) and HET KO ((**b**), *p* = 0.045). Abbreviations as in Figs 1 and 3.

We measured embryonic LV wall thickness using a 45-myocardial segments model (Supplementary Figure 2). A total of 56 mice at E18.5 and P0 were included (wildtype, n = 12; HET KO n = 16, HO KO n = 28, all on a C57BL/6J background, Fig. 6). In the wildtype mouse at E18.5 and P0, the septum is 36% thicker than the lateral wall at base and mid levels (levels 1–3: *P* = 0.0004, *P* = 0.002, *P* < 0.0001 by one-way ANOVA with Tukey correction) but not at the apex (levels 4,5: *P* = 0.397, *P* = 0.43). This basal-to-mid ASH pattern is more pronounced in HET and HO KO (all *P* < 0.0001, pooled stages). The HO KO mice had smaller overall LV cavity size compared to wildtype at birth and thinner absolute (un-indexed to LV size) LV myocardial segmental thicknesses (Fig. 6 and Table 1). LV chamber size (Supplementary Figure 3) in HO KO was smaller than in wildtype and HET KO: LV base-to-apical length was reduced in HO KO at E18.5 compared to wildtype (*P* = 0.02) and at P0 compared to both wildtype and HET KO (*P* < 0.0001 both); LV lumen diameter (antero-posterior) was reduced in HO KO compared to wildtype at P0 (*P* = 0.04). Data for the RV is provided in Supplementary Note 1.

## Discussion

Before the development of LVH there exists in humans a detectable subclinical HCM phenotype consisting of crypts (particularly multiple), elongation of the anterior mitral valve leaflet, increased LV apical trabecular complexity and smaller LV systolic volume, with the first two being the strongest predictors for sarcomere gene mutation carriage^20^.

It has long been argued that at least some aspects of this subclinical phenotype may have a developmental origin^8^ but proof of this was lacking. Here we report that in an animal model of HCM, there is a measurable embryological HCM phenotype.

Mutant HO and HET KO mice that lacked or expressed reduced levels of MyBP-C protein in the heart, are not structurally normal in utero as was previously thought^32^ but they have more crypts, abnormal patterns of trabeculation and the LVs of HO KO are smaller in size. While cardiac development is still able to proceed in spite of reduced or absent cMyBP-C levels, the cMyBP-C protein is essential for normal cardiac development.

Single myocardial crypts do occur in the general adult human population but multiple myocardial crypts are unusual in the healthy heart^33^,^34^. In the mouse, a reasonable and established model of human cardiac morphogenesis^35^, crypts are a normal and dynamic feature of the developing heart after ventricular septation supporting the literature descriptions by several authors of crypts being ‘congenital’^36^,^37^,^38^. In wildtype, crypts are most prevalent at E18.5 and typically disappear by birth, but crypts in HET and HO KO mutants are often multiple and persistent to birth. The location (predilection for RV superior and inferior insertion points) faithfully reflects adult human HCM crypts (see Supplementary Figure 4)^39^. We spotted only infrequent, singular crypts in the developing normal human heart, and although the number of hearts studied was small the data is in line with the murine findings. Since human myocardial crypts and late gadolinium enhancement by cardiac MRI due to plexiform fibrosis^40^ appear to share a similar predilection for RV superior and inferior insertion points^41^, further work should seek to explore the biomechanical or developmental factors potentially linking the two.

The mechanical environment during cardiogenesis directs stem cell differentiation^42^,^43^. At the time of myocardial migration and differentiation of pluripotent epicardium-derived cells (EPDCs)^44^, the heart tube has already begun to contract and is actively expressing *Mybpc3*^44^,^45^. It is reasonable to imagine that in mutants, the abnormal contractile properties of embryonic cardiomyocytes alter the tensile forces that converge on the RV insertion points, where muscle fibres are pulled apart creating deep crypts that the final myocardial compaction process after E18.5 cannot obliterate completely. Such crypts may therefore persist into the adult HCM heart.

AMVL elongation occurs in patients with overt and subclinical LVH^12^ and in adult HO *Mybpc3* KO mice with overt LVH^46^, but the mechanism remains elusive. Two hypotheses exist: the ‘congenital’ hypothesis proposes that AMVL elongation has its origin in the developing heart influenced by the sarcomere gene mutation; and the ‘acquired’ hypothesis, that the AMVL elongates postnatally in response to mechanical stretch from abnormal loading conditions. The fact that the AMVL is not elongated in our HET KO mice supports the latter hypothesis and fits with our previous work^11^ where we demonstrated AMVL elongation in all patients with overt HCM regardless of the presence of a sarcomere gene mutation by next generation sequencing. In addition these elongated leaflets in HCM patients have previously been shown to be histologically normal with no evidence of myxoid degeneration^47^.

It has been previously reported that HO KO mice on a Black Swiss background are born with an apparently normal cardiac phenotype^32^ only to develop increased heart-to-body-weight ratio by 2 weeks^32^ and reduced fractional shortening (heart failure) by 3–4 months^25^. Here, high-resolution imaging and more sophisticated analysis tools using mice on a C57BL/6J background show that at E18.5, HO KO exhibit significant basal septal hypertrophy compared to wildtype and by P0 they develop thinner LV walls and the LVs are smaller. Similar mice with homozygous *Mybpc3* gene mutations created by other researchers have been shown to develop dilated cardiomyopathy coupled with myocyte hyperplasia post-natally due to an extra round of postnatal cell division^48^.

Recent murine work has shown that doxycycline-induced inhibition of transgene expression of a different HCM-causing sarcomere gene mutation (α-myosin heavy chain gene^49^) only achieved LVH prevention when administered in utero, suggesting that the processes promoting LVH must be imprinted early in embryonic life. We show here embryonic patterning from HCM from the time of septation with altered cardiac developmental trajectory. It is for future studies to explore (potentially using imaging biomarkers similar to ours, as their surrogate end-points), whether primary preventative or therapeutic strategies for HCM are most effective when started sufficiently early: in utero for conditional transgenesis^49^; at birth for gene therapy^50^.

It has been hypothesized^25^ that the reduced cMyBP-C protein levels in KO might impair a molecular pathway through a deficit of interaction with a septum-specific protein, not yet identified, resulting in the development of age-dependent ASH in HET KO mice. Our data shows subtle ASH is the norm in wildtype mice at both E18.5 and P0. A theory could be that the basal septum is embryologically programmed towards ASH but normally kept in check by a healthy array of sarcomere gene products. Sarcomere gene mutations could be disinhibiting this embryological program, resulting in the florid ASH patterns characteristic of adult HCM.

Humans with HCM-causing heterozygous sarcomere gene mutations have previously been shown to have abnormally increased trabecular complexity^11^ resembling the situation seen in the HET KO mice at E18.5 compared to wildtype. Whilst this difference was not measurable at P0 (perhaps on account of the dramatic changes in LV wall structure and loading conditions at this stage), it is possible that the basis for abnormal trabecular formation is imprinted in utero and becomes manifest later. The hypotrabeculation measured in HO KO (for which no adult human corollary exists) is consistent with the generalised cardiac underdevelopment observed (thinner walls, smaller cavities, shorter mitral leaflets).

Limitations. First, we did not perform deep phenotyping of KO mice earlier than E18.5 as our more extensive phenotyping exercise in wildtype C57BL/6J and NIMR:Parkes (n = 108) suggested that crypt differences would be most significant at E18.5 and beyond. Second, it was necessary to use non-HREM imaging methods to study available human embryonic/fetal/neonatal hearts for crypts and while organ preparation and fixation protocols will have differed, we only examined hearts that had been fixed after diastolic arrest permitting the crypt survey. We added potassium chloride during heart preparation for HREM to encourage such diastolic arrest, but as this may have been incomplete and because the imaging plane for crypt survey in mice (HREM short-axis stack) differed from that published for adult humans by dynamic cardiac MRI (long axis cines where precise diastolic gating was possible), we set our HREM cut-off for defining a crypt at ≥30% instead of ≥50%. Third, some other histological aspects of the extended HCM phenotype^8^ like myocardial disarray, interstitial fibrosis and coronary arteriolar changes have not been investigated in this work. Fourth, although we attempted to measure abnormalities involving papillary muscles and chordae^47^ we found that accurate quantitative descriptions of these were not reliable in embryo hearts because of the density of myocardial trabeculae so no such data is presented. Fifth, we did not evaluate gender differences. Future work should be directed at elucidating the mechanism by which cMyBP-C protein haploinsufficiency in this model is altering the cardiac developmental trajectory, at studying mouse models with other types of *Mybpc3* and non-*Mybpc3* mutations, and to understand the impact of mouse genetic background on the severity of HCM phenotype expression.

In conclusion, adult human myocardial crypts first described in 1958^23^, have a congenital origin. By expressing exaggerated crypt formation^8^ patients with subclinical HCM may be recapitulating an embryonic cardiac state^51^,^52^, one that usually ceases to exist in the mouse after E18.5. Our animal data provides the first quantitative evidence that cMyBP-C has the ability to alter the cardiac developmental trajectory^53^,^54^. These novel developmental insights should stimulate further research in this area with the potential to improve our understanding of the pathogenesis and molecular mechanisms of HCM.

## Additional Information

**How to cite this article**: Captur, G. *et al*. The embryological basis of subclinical hypertrophic cardiomyopathy. *Sci. Rep.* **6**, 27714; doi: 10.1038/srep27714 (2016).

## Supplementary Material

Supplementary Information

Supplementary Video 1

Supplementary Video 2

Supplementary Video 3

Supplementary Video 4

## Figures and Tables

**Figure 1:**
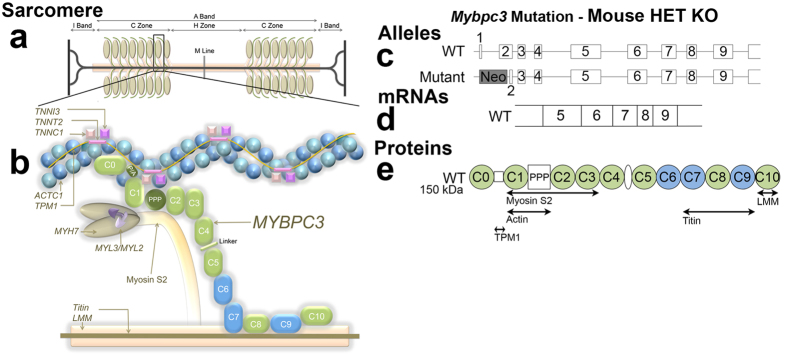
Schematic illustrating the mechanism of cMyBP-C haploinsufficiency in HET KO mice. (**a**) The cardiac sarcomere consists of thick-filament (myosin) and thin-filament proteins (actin, alpha cardiac muscle 1 [*ACTC1*], tropomyosin 1 alpha chain [*TPM1*], troponins I/C/T [*TNNI3, TNNC1, TNNT2*]) and titin. cMyBP-C is a sarcomeric protein located in the C zone of the A band that functions to stabilize sarcomere structure and regulate crossbridge cycling kinetics by controlling actomyosin interactions. Structurally wildtype cMyBP-C protein (**b**,**e**) consists of 12 domains of which 8 are immunoglobulin-like domains (pale green), and three are fibronectin domains (blue)^55^. Unique to cMyBP-C is a cardiac-specific C0 domain, a proline/alanine-rich (P/A) linker sequence between C0 and C1 and a regulatory motif between domains C1 and C2 that contains phosphorylation sites (PPP). The cMyBP-C interactome is illustrated in (**b**) and annotated in (**g**). All heterozygous (HET) HO mice (left panel) had a wildtype and a mutant recombinant allele (**c**) resulting from the targeted ablation of the transcription initiation site and exons 1–2. The final effect of the mutation in KO is that of a true protein haploinsufficiency predicted to produce a lower level of full length protein. LMM = light meromyosin; *MYH7* = β-myosin heavy chain cardiac muscle isoform; *MYL2* *=* myosin regulatory light chain 2 ventricular/cardiac muscle isoform; *MYL3* *=* myosin light chain polypeptide 3; Neo = neomycin resistance gene; WT = wildtype.

**Table 1:** HREM summary data from wildtype, HET KO and HO KO mice.

	Wildtype		HET KO		HO KO	
	E18.5	P0	E18.5	P0	E18.5	P0
No. of mice	8	4	7	9	18	10
LV length (mm)	2.33 ± 0.11	2.54 ± 0.06	2.25 ± 0.15	2.38 ± 0.12	2.18 ± 0.12‡	2.11 ± 0.06*^,^§
LV mid AP lumen (mm)	0.96 ± 0.10	1.01 ± 0.15	1.02 ± 0.04	0.91 ± 0.08	0.93 ± 0.07	0.86 ± 0.11‡
LV max. septal WT∞ (mm)						
Level-1	0.495 ± 0.059	0.598 ± 0.133	0.507 ± 0.077	0.568 ± 0.040	0.528 ± 0.044	0.566 ± 0.053
Level-2	0.481 ± 0.022	0.603 ± 0.102	0.527 ± 0.079	0.661 ± 0.039	0.570 ± 0.046‡	0.580 ± 0.068~
Level-3	0.566 ± 0.033	0.645 ± 0.051	0.577 ± 0.061	0.659 ± 0.067	0.573 ± 0.042	0.582 ± 0.075~
Level-4	0.520 ± 0.065	0.648 ± 0.135	0.527 ± 0.077	0.602 ± 0.039	0.587 ± 0.049	0.565 ± 0.066
Level-5	0.458 ± 0.026	0.585 ± 0.071	0.444 ± 0.061	0.577 ± 0.044	0.540 ± 0.064‖	0.532 ± 0.072
LV max. lateral WT≠ (mm)						
Level-1	0.339 ± 0.059	0.443 ± 0.099	0.401 ± 0.55	0.416 ± 0.054	0.415 ± 0.068	0.465 ± 0.068
Level-2	0.369 ± 0.033	0.470 ± 0.123	0.344 ± 0.039	0.434 ± 0.036	0.440 ± 0.074	0.423 ± 0.063
Level-3	0.378 ± 0.025	0.493 ± 0.079	0.351 ± 0.035	0.431 ± 0.036	0.402 ± 0.040	0.429 ± 0.062
Level-4	0.379 ± 0.021	0.525 ± 0.037	0.350 ± 0.051	0.419 ± 0.036	0.424 ± 0.040	0.440 ± 0.063
Level-5	0.428 ± 0.038	0.563 ± 0.104	0.387 ± 0.062	0.470 ± 0.041	0.435 ± 0.038	0.459 ± 0.064
No. of mice with:						
0 crypts	3	1	–	–	–	–
1 crypt	3	3	2	–	–	–
2 crypts	2	–	2	2	3	1
3 crypts	–	–	2	4	5	2
4 crypts	–	–	–	3	5	–
5 crypts	–	–	–	–	2	3
6 crypts	–	–	1	–	1	2
7 crypts	–	–	–	–	2	1
8 crypts	–	–	–	–	–	1
No. of mice with ≥1 crypt (%)	5 (63)	3 (75)	7 (100)	9 (100)	18 (100)‡	10 (100)
No. of mice with ≥2 crypts (%)	2 (25)	0 (0)	5 (71)	9 (100)†	18 (100)*	10 (100)*
Crypt depth (mm)	0.187 ± 0.17	0.236 ± 0.16	0.184 ± 0.11	0.249 ± 0.02	0.224 ± 0.30	0.244 ± 0.02
Crypt depth:WT ratio (% ± se)	38.2 ± 4.1	49.0 ± 8.5	55.6 ± 1.8	55.4 ± 0.8	49.7 ± 0.5	52.7 ± 0.5
AMVL (mm)						
Length	0.472 ± 0.064	0.480 ± 0.072	0.451 ± 0.065	0.458 ± 0.060	0.365 ± 0.032*^,^‖	0.399 ± 0.028
Basal thickness	0.099 ± 0.024	0.094 ± 0.023	0.081 ± 0.018	0.062 ± 0.023	0.071 ± 0.024‡	0.047 ± 0.009†
PMVL (mm)						
Length	0.249 ± 0.031	0.333 ± 0.078	0.247 ± 0.053	0.326 ± 0.056	0.238 ± 0.019	0.273 ± 0.031
Basal thickness	0.065 ± 0.012	0.052 ± 0.013	0.046 ± 0.013	0.053 ± 0.014	0.060 ± 0.011	0.041 ± 0.013

The segment-by-segment LV wall thickness data reproduced in Fig. 6 is organised in this table to permit comparisons of septal versus lateral wall thicknesses at the various levels. AMVL = anterior mitral valve leaflet; AP = anteroposterior; HREM = high-resolution episcopic microscopy; No./nos = number/s; PMVL = posterior mitral valve leaflet; se = standard error. All values are expressed as mean ± standard deviation (s.d.) unless otherwise stated.

^∞^Segments 1, 6, 7, 8 and 9 are considered in the septal group.

^≠^Segments 2, 3, 4 and 5 are considered in the lateral group.

^*^Significantly different from wildtype same stage, *p* < 0.001.

^†^Significantly different from wildtype same stage, *p* < 0.01.

^‡^Significantly different from wildtype same stage, *p* < 0.05.

^§^Significantly different from HET KO same stage, *p* < 0.001.

^‖^Significantly different from HET KO same stage, *p* < 0.01.

^~^Significantly different from HET KO same stage, *p* < 0.05.
