# Supplementary material for: The embryological basis of subclinical hypertrophic cardiomyopathy
Source: Sci Rep. 2016 Jun 21;6:27714. doi: 10.1038/srep27714 (PMC4914973; doi:10.1038/srep27714)
Supplement: Supplementary Information [file srep27714-s1.doc]

SUPPLEMENTARY INFORMATION FOR:

**The embryological basis of subclinical hypertrophic cardiomyopathy**

Gabriella Captur^1^ MD PhD MRCP MSc, Carolyn Y Ho^2^ MD PhD, Saskia Schlossarek^3,4^ PhD, Janet Kerwin^5^ PhD, Mariana Mirabel^6^ MD PhD, Robert Wilson^7^ PhD, Stefania Rosmini^8^ MD, Chinwe Obianyo^8^ MD MSc, Patricia Reant^9^ MD PhD, Paul Bassett^10^ MSc, Andrew C Cook^8^ MD, Susan Lindsay^5^ PhD, William J McKenna,^8^ MD, FACC, FRCP, Kevin Mills PhD,^1^ Perry M Elliott^8,11^ MD, FRCP, FESC, FACC, Timothy J Mohun^7^ PhD, Lucie Carrier^3,4^ PhD, James C Moon^8,11*^ MD, MBBS, MRCP.

1. UCL Biological Mass Spectrometry Laboratory, Institute of Child Health and Great Ormond Street Hospital, 30 Guilford Street, London, UK

2. Cardiovascular Division, Brigham and Women’s Hospital, Boston MA, USA

3. Department of Experimental Pharmacology and Toxicology, Cardiovascular Research Center, University Medical Center Hamburg-Eppendorf, Hamburg, Germany

4. DZHK (German Center for Cardiovascular Research), partner site Hamburg/Kiel/Lübeck, Hamburg, Germany

5. Institute of Genetic Medicine, Newcastle University, Newcastle, UK

6. INSERM U970, Paris Cardiovascular Research Center—PARCC, Paris, France

7. The Francis Crick Institute Mill Hill Laboratory, The Ridgeway, Mill Hill, London, UK

8. UCL Institute of Cardiovascular Science, University College London, Gower Street, London, UK

9. University of Bordeaux, CHU de Bordeaux, CIC1401, Bordeaux, France

10. Biostatistics Joint Research Office, University College London, Gower Street, London, UK

11. Barts Heart Center, The Cardiovascular Magnetic Resonance Imaging Unit and The Center for Rare Cardiovascular Diseases Unit, St Bartholomew’s Hospital, West Smithfield, London, UK

***CORRESPONDENCE**

Prof James C Moon

Institute of Cardiovascular Science,

University College London,

Gower Street,

London WC1E 6BT, UK

E-mail: j.moon@ucl.ac.uk Phone No: +44 2034563081 Fax No: +0203 456 3086

**SUPPLEMENTARY NOTES**

**Note S1. Right ventricular wall thickness and size in wildtype, HET KO and HO KO**

Right ventricular (RV) free wall thickness at the 9 o’clock position was measured using linear caliper tools in OsiriX at three levels: basal, mid and apical. A total of 56 mice at E18.5 and P0 (wildtype, n = 12; HET KO n = 16, HO KO n = 28, all on a C57BL/6J background) were included in this analysis. Analysis showed that the RV basal free wall was thinner in HO KO compared to wildtype at P0 (*p* = 0.037) but other measurements were similar between groups.

**SUPPLEMENTARY TABLES**

**Table S1.** **Summary of human embryo, fetal and neonatal imaging datasets.**

| **No.** | **Developmental**  **Stage** | **Imaging**  **Modality** | **Image**  **Resolution** (μm) | **Crypt**  (Y/N) | **Data Source** |
| --- | --- | --- | --- | --- | --- |
| 1 | CS20 | HREM | 2 | 0 | HBDR |
| 2 | CS21 | OPT^1^ | 20 | 1 | HBDR |
| 3 | 9 pcw | MRI | 70 | 1 | HBDR |
| 4 | 19 pcw | Micro-CT | 3 | 0 | ICH UCL |
| 5 | Neonatal | MRI | 340 | 0 | ICL UCL |

CS = Carnegie stage, CT = computerised tomography, HBDR = Human Developmental Biology Resource, HREM = high-resolution episcopic microscopy, ICH UCL = Institute of Child Health University College London, MRI = magnetic resonance imaging, N = neonatal, OPT = optical projection tomography, pcw = post-coital week.

**Table S2.** **Distribution of wildtype, HO/HET *Mybpc3*-targeted KO mice per littermate.**

| **Littermate**  **#** | **Wildtype**  **E18.5** | **Wildtype P0** | **HET KO**  **E18.5** | **HET KO P0** | **HO KO E18.5** | **HO KO P0** |
| --- | --- | --- | --- | --- | --- | --- |
| 1 | 8 |  |  |  |  |  |
| 2 |  | 4 |  |  |  |  |
| 3 |  |  | 7 |  |  |  |
| 4 |  |  |  | 9 |  |  |
| 5 |  |  |  |  | 8 |  |
| 6 |  |  |  |  | 10 |  |
| 7 |  |  |  |  |  | 10 |
| Total | 8 | 4 | 7 | 9 | 18 | 10 |

KO = knock-out, HCM = hypertrophic cardiomyopathy, HO = homozygous, HET = heterozygous.

**SUPPLEMENTARY FIGURES**

**Supplementary Figure 1 Compaction data for the right ventricle in wildtype, HET KO and HO KO.** Solid lines represent group means. Ribbons represent upper and lower 95% confidence limits. Trabeculation in the right ventricle (RV) was measured using fractal analysis^2^ in wildtype, HET KO and HO KO mice (n = 56 at E18.5 [**a**] and P0 [**b**]; wildtype, n = 12; HET n = 16, HO n = 28). We show how at E18.5 (**a**) the RV of HET KO is more noncompacted compared to both wildtype and HO KO (*p* < 0.0001 both, by ANCOVA). By contrast the RV of HO KO was less noncompacted than wildtype and HET KO across both stages (*p* < 0.0001 all).

**
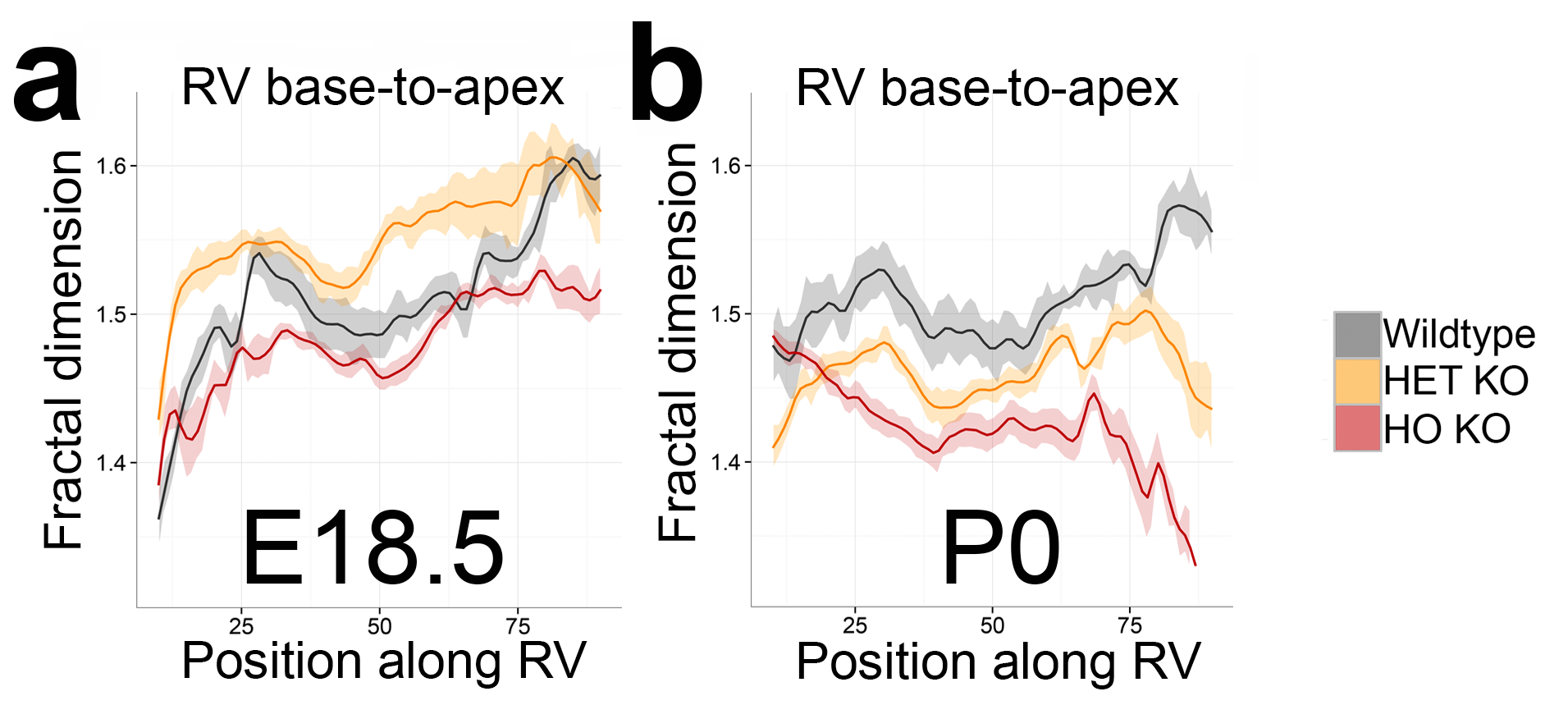
**

**Supplementary Figure 2 Semi-automated method for wall thickness measurement of HREM data.** (**a**) From the user-defined cardiac centroid 9 radial spokes are emanated at 40**°** intervals, transecting the LV walls from which linear caliper measurements are then extracted. Trabeculae were manually excluded from the wallt thickness measurement. Where the moderator band or right ventricular septomarginal trabeculum were recognized, these were excluded from septal wall thickness measurements in line with established guidance.^3^ (**b**) Radial grids were applied at 5 levels from LV base to apex generating a total of 45 myocardial segments (9 per level) that were then rendered into bullseye plots using MATLAB**®**. Segments 1, 6, 7, 8 and 9 were counted as ‘septal’ and segments 2, 3, 4 and 5 as ‘lateral’ for each of the 5 ventricular levels


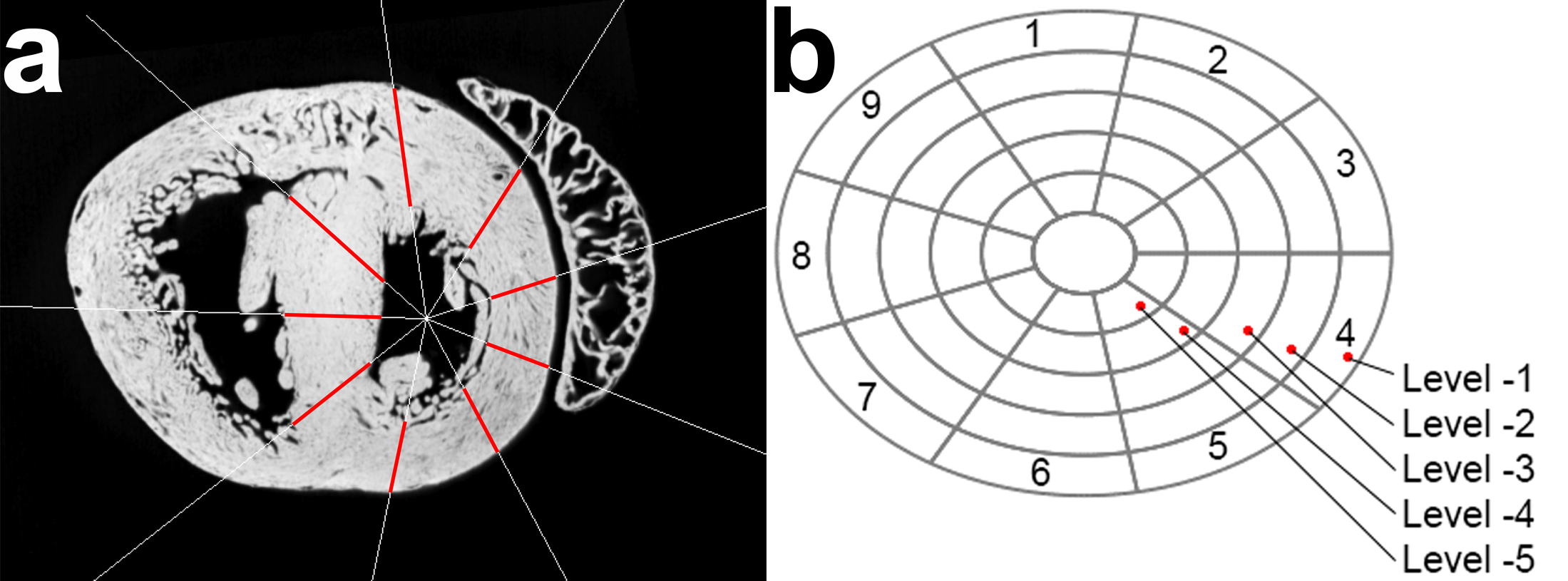


**Supplementary Figure 3 Example measurements of left ventricular length and anteroposterior luminal dimensions.** Example images from a wildtype mouse at E18.5. (**a**) Whole heart volume-rendered 3D reconstruction in OsiriX. (**b**) Eroded coronal image showing caliper measurement of LV length from the mitral annular plane to the epicardial surface of the LV apex. (**c**) Eroded short axis view showing caliper measurement of LV mid ventricular anteroposterior internal luminal diameter at the level of the papillary muscles. Scale bars, 0.5 mm.


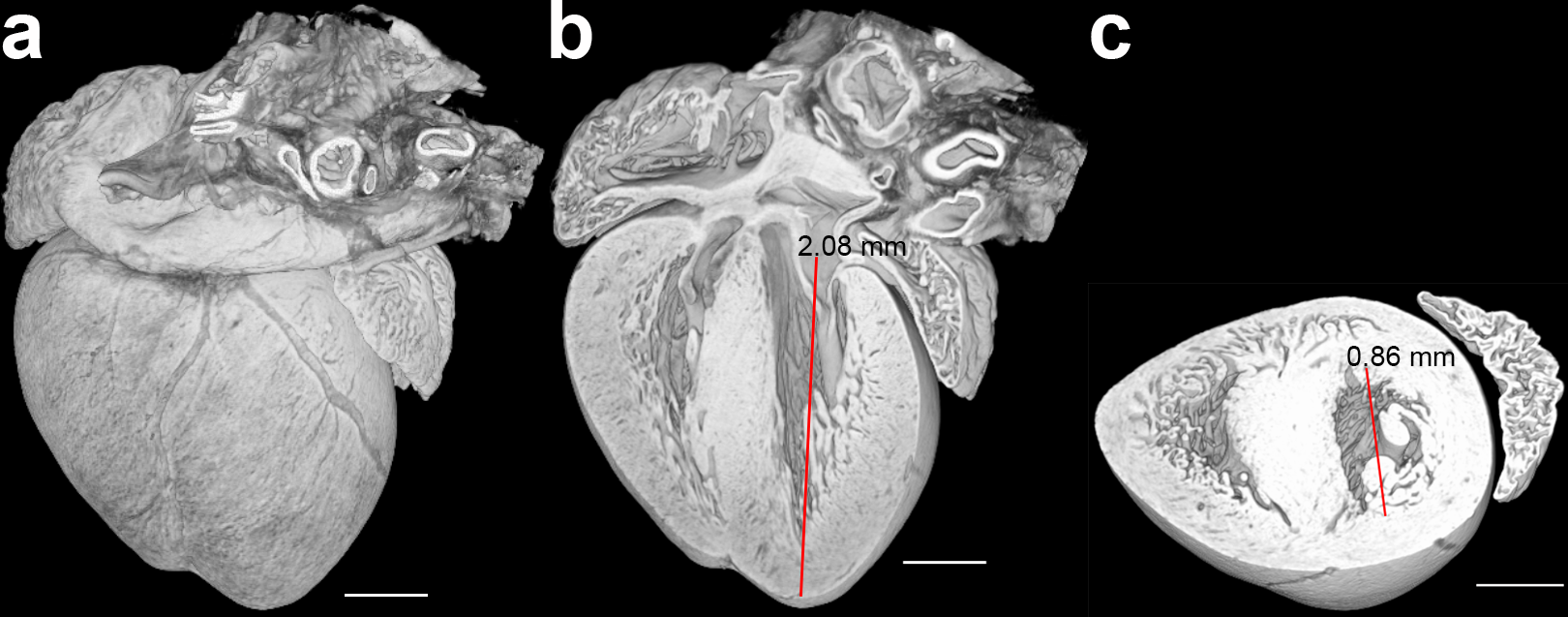


**Supplementary Figure 4 Crypts in adult human hearts.**

(a) Cardiovascular magnetic resonance (CMR) cines pertaining to twelve adult patients with HCM caused by a truncating *MYBPC3* mutation (c.772G>A transition, NM_000256.3). Ten patients exhibited at least one crypt (*); two patients had no crypts (bottom right). The CMR protocol was approved by the institutional review boards of the respective US and EU centers. Written informed consent was obtained from all participants according to the ethics committee guidelines at the respective academic centers. All 12 patients pertaining to 10 unrelated families had consented to blood sample collection at initial evaluation and genomic DNA had been isolated from peripheral blood lymphocytes, using standard methodology. CMR was performed using 1.5 and 3-Tesla (T) magnets from different manufacturers. (b) A basal inferior crypt as seen in a long and short-axis left ventricular HREM view in wildtype (WT) mouse at E18.5 that is strikingly similar to the basal inferior crypt in a healthy adult human heart imaged by CMR. Scale bar, 0.5mm. NIMR = National Institute of Medical Research.

**
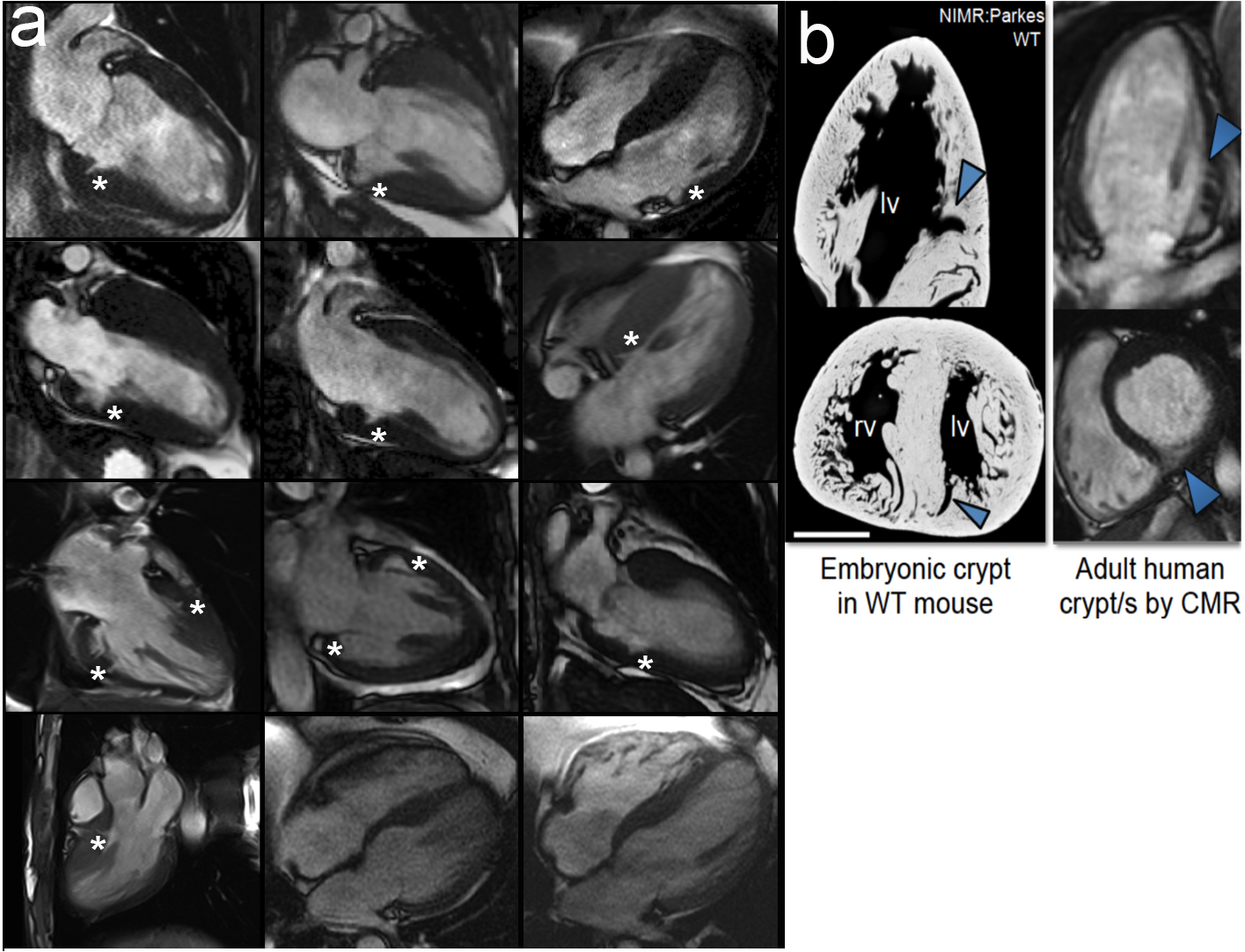
**

**SUPPLEMENTARY VIDEOS**

Video S1.

(a) Volumetric model of left ventricular lumen derived from HREM data of a HO KO embryonic heart (C57BL/6J background) at P0. Initially viewing each free wall side, the digital cast rotates to show the septal wall surface and also the multiple deep myocardial crypts that appear as finger-like projections (red arrows). (b) Virtual HREM image stack of the same heart, digitally eroding from base to apex, revealing the same deep myocardial crypts as in (a) but in the short-axis view.

Video S2.

Black and white 2D movie of a raw HREM stack, from base to apex, showing a different HO KO mouse heart at P0. Red arrows point to the location of three deep myocardial crypts.

Video S3.

Black and white 2D movie of a raw HREM stack, from base to apex, showing a wildtype mouse heart at P0 lacking deep myocardial crypts.

**REFERENCES FOR SUPPLEMENTARY MATERIAL**

1. Kerwin, J. *et al.* 3 dimensional modelling of early human brain development using optical projection tomography. *BMC Neurosci.* **5,** 27 (2004).

2. Captur, G. *et al.* Morphogenesis of myocardial trabeculae in the mouse embryo. *J. Anat.* **1,** 100–105 (2016).

3. Ho, S. Y. & Nihoyannopoulos, P. Anatomy, echocardiography, and normal right ventricular dimensions. *Heart* **92 Suppl 1,** i2–13 (2006).
